# The family physician’s guide to the private sector galaxy

**DOI:** 10.4102/safp.v65i1.5682

**Published:** 2023-02-17

**Authors:** Sheena Mathew, Shane D. Murphy

**Affiliations:** 1 Private Practice, Blouberg, South Africa; 2 Division of Family Medicine, University of Cape Town, Cape Town, South Africa; 3 Clinical Services, Mediclinic Pietermaritzburg, South Africa

## Introduction

As dramatic as the title might sound, for most newly qualified family physicians (FPs), the thought of entering the private sector can bring about the same sense of unfamiliarity, uncertainty, and fear as one would expect with embarking on an extraterrestrial venture. This piece aims to provide a guide for navigating this ‘road less travelled’ and hopes to inspire more colleagues to make their way into the private sector so that family medicine (FM) can firmly establish itself as a pivotal part of the private healthcare landscape. The public sector structure (of primary, district, and tertiary levels of care) lends itself towards the placement of FPs in leadership and governance roles within the healthcare system. Comparatively, the private sector is limited to primary (e.g., medical centres) and tertiary (e.g., private hospitals) healthcare services, making it more challenging to clearly position ourselves.

## Being a family physician

Training in FM follows a comprehensive and integrated design. The FP is not only an expert care provider, but also a capacity builder, clinical trainer, consultant, clinical governance leader, and champion of community-oriented primary care.^1^ We must reckon with our professional integrity – are these dimensions lost as we venture into the private sector? The answer is a clear no!

## Roles and opportunities

The private sector holds many untapped avenues for the FPs to leverage their training towards the betterment of society. Roles include joining partners in a practice, functioning as a specialist consultant for medical schemes (healthcare funders) or non-governmental and non-profit organisations, as well as several managerial roles (dedicated entirely to clinical governance and health system strengthening). Furthermore, the looming healthcare reform envisaged by the National Health Insurance scheme^2^ represents exciting opportunities where the six roles of the FP will play an important role in providing redress to South Africa’s outdated (and unsustainable) bipartite model of healthcare.

## Getting started in private practice

It is useful to clearly describe what you want to achieve. Some questions to ask yourself before venturing into private practice include:

- How do you envision practising FM principles within your community?
- What are your main work-related priorities (i.e., flexibility, income level, diversity versus focused practice, scope of practice, managerial involvement, clinical expertise, skills development, wanting office hours)?
- What are the risks and benefits of solo versus partnership/associations?
- What financial resources (loans or saved capital) will you need to cover at least 6 months of initial overhead costs?
- What will your scope of practice be (outpatient, hospital based, family practice, specialised services for women’s health/mental health/HIV/palliative care, ultrasound services)?
- What is the sociodemographic profile of your intended practice area (young families, elderly, students) and their healthcare needs?

Once you have clearly defined your practice (and have decided to embark on establishing a private practice), the next step involves putting together a business plan that clearly outlines the following:

- Obtain practice registration with Board of Healthcare Funders – FM Specialist (015, not 014).
- Develop a budget and tabulate the financial resources available to you (combined resources if partnership/association) as well as those that you might need.
- Identify a practice location and weigh up rental options versus purchasing a property.
- Explore electronic medical billing, book-keeping, and record-keeping solutions to find one that works for you.
- Ensure that you have appropriate information technology infrastructure services (such as fibre, telecom systems, back-up power and data security).
- Use predefined checklists to ensure that you have the required basic and specialised medical equipment to safely run your practice.
- Ensure that your staff are an asset to your practice by educating them about a family physician’s scope of practice and holistic care approach, so they can effectively administrate your practice as well as educate potential patients that call with queries.
- Identify and harness education and marketing strategies (e.g., website with online appointments, meet and greet, Google ads, educational blogs, and personalised branding and logo).

## The next steps

It is clear that there are multiple factors to consider when venturing into the private sector – do not go alone! Join a community such as the South African Academy of Family Physician Private Sector Forum,^3^ which has been created to advocate for the private sector needs, or the Next5 initiative^4^ where recently qualified FPs can share lessons learnt and enjoy the mentorship of experienced FPs.

## References

[1] Mash R, Von Pressentin K. Family medicine in South Africa: Exploring future scenarios. South Afr Fam Pract. 2017;59(6):224–227. 10.1080/20786190.2016.1272231

[2] Murphy SD, Moosa S. The views of public service managers on the implementation of National Health Insurance in primary care: A case of Johannesburg Health District, Gauteng Province, Republic of South Africa. BMC Health Serv Res. 2021;21(1):969. 10.1186/s12913-021-06990-4 34521399 PMC8439954

[3] The South African Academy of Family Physicians. Private Sector Forum. Services [homepage on the Internet]. 2021 [cited 2022 Nov 21]. Available from: https://saafp.org/next5/

[4] The South African Academy of Family Physicians. The Next5 Initiative. Services [homepage on the Internet]. 2021 [cited 2022 Nov 21]. Available from: https://saafp.org/privatefamilyphysicianforum/

